# Detection of Bleeding Events in Electronic Health Record Notes Using Convolutional Neural Network Models Enhanced With Recurrent Neural Network Autoencoders: Deep Learning Approach

**DOI:** 10.2196/10788

**Published:** 2019-02-08

**Authors:** Rumeng Li, Baotian Hu, Feifan Liu, Weisong Liu, Francesca Cunningham, David D McManus, Hong Yu

**Affiliations:** 1 College of Information and Computer Science University of Massachusetts Amherst Amherst, MA United States; 2 Department of Computer Science University of Massachusetts Lowell Lowell, MA United States; 3 Department of Quantitative Health Sciences University of Massachusetts Medical School Worcester, MA United States; 4 Department of Veterans Affairs Center for Medication Safety Hines, IL United States; 5 Cardiology Division Department of Medicine University of Massachusetts Medical School Worcester, MA United States; 6 Center for Healthcare Organization and Implementation Research Bedford Veterans Affairs Medical Center Bedford, MA United States

**Keywords:** autoencoder, BiLSTM, bleeding, convolutional neural networks, electronic health record

## Abstract

**Background:**

Bleeding events are common and critical and may cause significant morbidity and mortality. High incidences of bleeding events are associated with cardiovascular disease in patients on anticoagulant therapy. Prompt and accurate detection of bleeding events is essential to prevent serious consequences. As bleeding events are often described in clinical notes, automatic detection of bleeding events from electronic health record (EHR) notes may improve drug-safety surveillance and pharmacovigilance.

**Objective:**

We aimed to develop a natural language processing (NLP) system to automatically classify whether an EHR note sentence contains a bleeding event.

**Methods:**

We expert annotated 878 EHR notes (76,577 sentences and 562,630 word-tokens) to identify bleeding events at the sentence level. This annotated corpus was used to train and validate our NLP systems. We developed an innovative hybrid convolutional neural network (CNN) and long short-term memory (LSTM) autoencoder (HCLA) model that integrates a CNN architecture with a bidirectional LSTM (BiLSTM) autoencoder model to leverage large unlabeled EHR data.

**Results:**

HCLA achieved the best area under the receiver operating characteristic curve (0.957) and F1 score (0.938) to identify whether a sentence contains a bleeding event, thereby surpassing the strong baseline support vector machines and other CNN and autoencoder models.

**Conclusions:**

By incorporating a supervised CNN model and a pretrained unsupervised BiLSTM autoencoder, the HCLA achieved high performance in detecting bleeding events.

## Introduction

### Background and Significance

Bleeding is defined as the escape of blood from the circulatory system (arteries and veins) due to trauma, anatomic malformation, bleeding disorder, medications, and aging. Bleeding events include symptoms like reddening or darkening of urine or stools, bleeding of gums, blood blisters, bruises, and vomiting of blood. Studies show that high incidences of bleeding events are associated with cardiovascular disease in patients on anticoagulant therapy [1-9], which has contributed to its standing as the most-frequent adverse drug events (ADEs) [1,3-9]. Anticoagulants are considered a high-alert medication by the Institute for Safe Medication Practices because of the potential severity of anticoagulant-related bleeding. In a study on patients receiving oral anticoagulant therapy, major bleeding occurred at a rate of 7.22 per 100 person-years and fatal bleeding occurred at a rate of 1.31 per 100 person-years, with a case-fatality rate of 13.4% for major bleeding [3]. Adverse health outcomes resulting from bleeding include poor functional status, myocardial infarction, heart failure, stroke, and even death [3-9]. Prompt and accurate detection of bleeding events is essential to prevent such adverse health outcomes and improve drug-safety surveillance and pharmacovigilance.

Bleeding events are frequently not recorded in the structured fields and are buried in the electronic health record (EHR) notes [10]. Manual abstraction is prohibitively expensive. Rapid, accurate, and automated detection of bleeding events in EHR notes may have significant cost and logistical benefits over manual detection. Therefore, this study aimed to develop natural language processing (NLP) approaches to automatically detect bleeding events in EHR notes.

NLP approaches have demonstrated increasing utility in clinical text mining in recent years [11-14]. Deep neural network methods have recently achieved new state-of-the-art performance in a wide range of NLP tasks [15-17]. In this study, we explored deep learning models and compared them with the strong traditional machine-learning classifiers (eg, support vector machines [SVM]).

Two architectures of deep neural networks relevant to this work include convolutional neural network (CNN) [18] and recurrent neural network (RNN) with its variants of long short-term memory (LSTM) [19] and gated recurrent unit [20]. Both architectures have demonstrated advantages in text-processing tasks. The CNN models use layers with convolutional filters that are applied to local features [18] and therefore are able to capture local relationships between neighboring *w*-gram words in a sentence, but are less efficient for long-distance dependencies. In contrast, the LSTM models [19] are designed to learn long-term dependencies by maintaining an internal state, which represents the memory cell of the LSTM neuron. Thus, the LSTM models are able to memorize information for a longer duration than the CNN models. Bleeding events can be inferred by local context. Therefore, we chose CNN as the major model of our architecture, but leveraged the LSTM model to learn sentence-level representation. Our CNN model differs from the previous neural network models in that we deployed an autoencoder neural network [21] as an unsupervised learning algorithm to learn a latent representation from unlabeled sentences in order to help improve CNN performance. Specifically, we propose the hybrid CNN and LSTM (HCLA) autoencoder model, which employs a CNN model that is integrated with a bidirectional LSTM (BiLSTM)-based autoencoder model to classify whether a sentence contains a bleeding event.

The knowledge-acquisition bottleneck problem presents a unique challenge in clinical NLP. Unlike data collection in the open domain, crowdsourcing methods (eg, Amazon Mechanic Turks [22]) cannot be easily applied to medical domain data collection due to privacy concerns. Annotation by medical professionals is expensive and time consuming, and annotated data in the clinical domain are typically limited. Because our HCLA model leverages a large number of unlabeled EHR notes, our results demonstrate that domain-specific features learned through such an autoencoder can effectively improve the supervised learning performance, despite the small amount of the training data.

### Related Works

Existing work in automated bleeding detection mainly involves detection and classification of bleeding for wireless capsule endoscopy images. Neural network methods are also employed for such image detection [23,24]. In addition, previous studies have assessed detection of bleeding events in outcome studies by using health registers [25]. However, studies on the detection of bleeding events in EHR notes are lacking.

The proposed model is based on neural network models that learn feature representations for sentence-level classification. Related work includes the CNN models that first made a series of breakthroughs in the computer vision field and subsequently showed excellent performance in NLP tasks such as machine translation [26], sentence classification [27,28], and sentence modelling [29]. Autoencoders were originally proposed to reduce the dimensionality of images and documents [21] and were subsequently applied to many NLP tasks such as sentiment analysis [30], machine translation [31], and paraphrase detection [32].

Neural network models have been applied to the clinical data-mining tasks. Gehrmann et al [33] applied CNNs to 10 phenotyping tasks and showed that they outperformed concept extraction-based methods in almost all tasks. CNN was used to classify radiology free-text reports and showed an accuracy equivalent to or beyond that of an existing traditional NLP model [34]. Lin et al [35] also used a CNN model to identify the International Classification of Diseases, Tenth Revision, Clinical Modification, diagnosis codes in discharge notes and showed outstanding performance compared with traditional methods; they also showed that the convolutional layers of the CNN can effectively identify keywords for use in the prediction of diagnosis codes. Since our annotated data are relatively small, we expanded the CNN model by integrating it with an LSTM-based autoencoder. Tran et al [36] developed two independent deep neural network models: one based on CNNs and the other based on RNNs with hierarchical attention for the prediction of mental conditions in psychiatric notes; their study showed that the CNN and RNN models outperformed the competitive baseline approaches. Furthermore, a previous study used semisupervising learning methods such as learning from positive and unlabeled examples [37] and the anchor-and-learn method [38], for which traditional machine-learning algorithms like expectation–maximization and SVM can be used to build classifiers.

## Methods

### Models

The EHR notes used in this study were provided by the University of Massachusetts Medical School. Our study was approved by the Institutional Review Board. All the EHR notes were deidentified.

**Figure 1:**
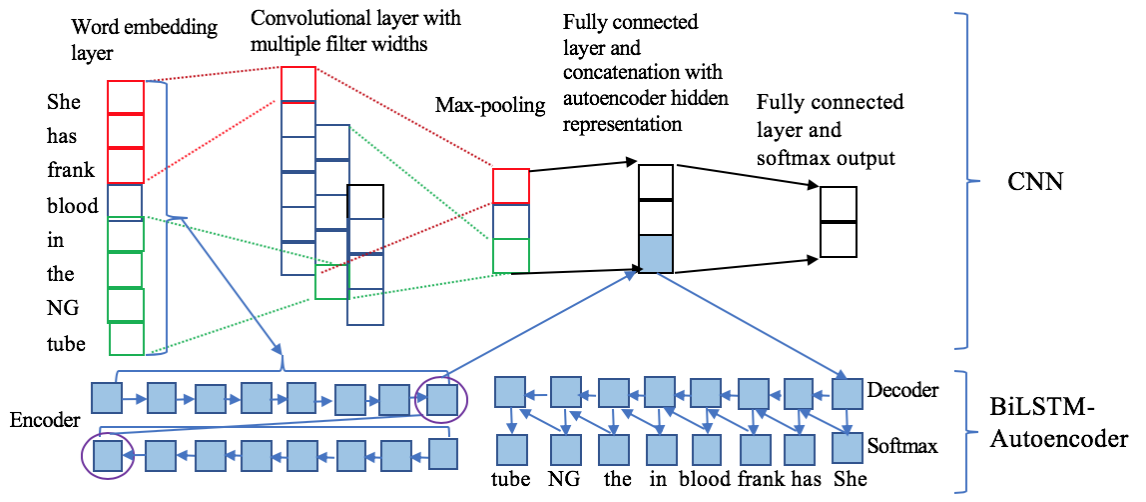
The hybrid convolutional neural network (CNN) and long short-term memory (LSTM) autoencoder (HCLA) model architecture with two major components: the CNN model and the bidirectional LTSM (BiLSTM)-based autoencoder. NG: nasogastric.

Our HCLA model integrates a supervised CNN architecture and an unsupervised BiLSTM-based autoencoder. Given a sentence as input, we trained a classifier to determine whether the sentence contains a bleeding event. Figure 1 illustrates the architecture of our model. After passing the sentence through the CNN model and the BiLSTM-based autoencoder simultaneously, the HCLA model generates two separate representations for the input sentence: local features encoded by the CNN model and global features encoded by the autoencoder. A softmax function was then used to determine whether the input contained a bleeding event.

### Bidirectional Long Short-Term Memory-Based Autoencoder

An autoencoder is a neural network that typically has three layers: an input layer, a hidden (encoding) layer, and a decoding layer. Through the encoding process, the inputs are compressed into a hidden representation, which is then used to reconstruct the input back in the decoding process.

A BiLSTM-based autoencoder has two major parts: encoder and decoder. During the encoding phase, an LSTM is used to scan the input *X*={*x*_*1*_, *x*_*2*_... *x*_*i*_... *x*_*n*_} in a sequential order. Each time, it takes a word *x*_*i*_ with *e*_*i*_ as its embedding and the hidden representation *h*_*i*__–1_ generated at the previous step as the input to generate the representation 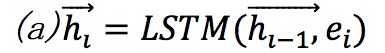
for the current step.

The final is 
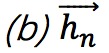
the representation of the input sentence. A BiLSTM model uses the same LSTM to scan the input sentence again in reverse and obtains another representation 
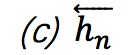
, so that the input is encoded as the concatenated hidden representation 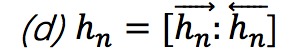, *h*_*n*_ ∈ *R*^*k*^, where *k* is a predefined dimensionality.

The decoder is another LSTM layer. The hidden representation is fed to the decoder LSTM layer to reconstruct the input words. First, we set *h* *'*_0_=h_n_ to repeat the following steps until the input is reconstructed:


*h*
*'*_*i*_=LSTM(
*h*
*'*_*i*__–1,_ e
_*i*__–1_)

o
_i_=
*Wh*
*'*_*i*_+
*b*

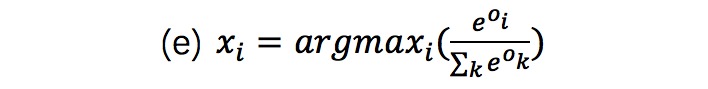

e_i_ = emb(x_i_)

The LSTM takes the *h* *'*_*i*__–1_ that is the hidden state of the previous step and e_*i*__–1_ that is the word generated in the previous step as input and updates *h* *'*_*i*__–1_ to *h* *'*_*i*_. Subsequently, *h* *'*_*i*_ is passed through a softmax layer to generate the word at *i*^th^ step *x*_*i*_*.*

After training by the abovementioned procedures, the hidden representation 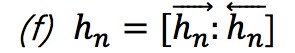
is obtained as a condensed and better representation of the sentence.

### Convolutional Neural Networks

The CNN model takes a sequence of words as input and outputs a fixed-length low-density vector as a representation of the input. Words are first represented using their embeddings, which can be learned during training or loaded from pretrained models. We will report how we set the embeddings for each of our specific models in the Models subsection. The sentence subsequently becomes a matrix whose dimensions are decided by the word numbers of the input and the dimensions of word embeddings. Convolutional layers of different sizes scan the matrix to generate a new and dense representation of the input. The newly generated representations are further projected to a fixed-length vector through max pooling as the final representation of the input. We adapted the architecture of an open-domain CNN model [27] with the following components:

1. Input layer: The input is a sequence of tokens *X*={*x*_*1*_, *x*_*2*_... *x*_*i*_... *x*_*n*_} where *n* denotes the length of the sentence. Token *x*_*i*_ is associated with a d-dimensional embedding *e*_*i*_*, e*_*i*_={*e*_*i*_^*1*^, *e*_*i*_^*2*^... *e*_*i*_^*d*^}. Therefore, the input is represented as a feature map of dimensionality n×d.
2. Convolutional layer: A convolutional operation with a filter sliding over the input is applied for local feature learning. Given the input sentence *X*={*x*_*1*_, *x*_*2*_... *x*_*n*_} (zero padded if necessary), a feature *c*_*i*_ can be learned from a window of words [x_i:i+k–1_] as *c*_*i*_= *f* (*w* [x_i:i+k–1_]+ *b*), where *w* is the convolutional weights *w* ∈ *R*^*d*^^*×*^^*kd*^, bias b ∈ *R*^*d*^, *f* is the nonlinear activation function (we used a hyperbolic tangent in our experiments), and *k* is the filter width. In our model, we used three filters of width 3, 4, and 5, and the number of filters used was 10.
3. Max-pooling layer: A max operation is applied to the result of each filter to keep the most-salient information and reduce dimensionality 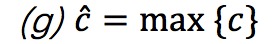. The outputs of the three filters after max pooling are concatenated in this framework.
4. Concatenation layer: After the max-pooling layer was fully connected, at this step, we concatenated the final hidden representation 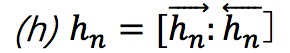
of the encoder layer obtained from the BiLSTM-based autoencoder approach, as described in the above Bidirectional Long Short-Term Memory-Based Autoencoder subsection.
5. Softmax layer: Another fully connected layer and softmax operation was applied for the prediction. The cut-off point of 0.5 was used to convert the predicted probability to a binary outcome with regard to whether the sentence contains a bleeding event.

## Results

### Experimental Datasets

We expert annotated a corpus of 878 longitudinal EHR notes of patients with cardiovascular events. This corpus contains 76,577 sentences and 562,630 word-tokens. Each note was annotated by at least two physicians. The interannotation agreement (Cohen *k*) among the annotators was *k*=0.9182.

We preprocessed the data and removed duplicate sentences. In addition, we removed sentences with length <5 word-tokens, as those were mostly incomplete sentences or sentence fragments. The remaining 48,628 sentences were used for training and validation. Textbox 1 presents the representative sentences in our annotated corpus.

Although we had a total of 878 annotated notes, only 291 notes mentioned bleeding events, from which we identified a total of 1451 sentences. These 1451 sentences were considered as “positive” data for training. From the 291 notes, a total of 285 sentences that mentioned bleeding events, but were annotated as negation. Those sentences presented as “harder” examples for our NLP systems, and we included them in the “negative” data for training.

We performed downsampling to include equal number of positive and negative data for training and validation. Since we had included 285 sentences that contain negated bleeding events, 1166 negative sentences were randomly selected from the remaining negative sentences in the 291 notes, as those sentences were more challenging for an NLP system to identify than sentences from the notes that did not contain a bleeding event. Although we did not include any sentence from the 587 annotated notes that did not contain a bleeding event, the 32,704 sentences from those notes were included to train the autoencoder on feature representation.

Of the combined 2902 positive and negative sentences (50% each), we randomly selected 1 of 10 sentences as the testing data, and the other 9 sentences were used for model training. By setting aside the testing data, we trained the BiLSTM-based autoencoder model on all the remaining sentences of the 878 notes, with a total of 48,338 sentences after preprocessing.

An NLP system that is trained for downsampling may not perform well for the real-world data; in our case, the positive and negative data were highly unbalanced (only 2.9% of annotated sentences contained a bleeding event). To accurately evaluate the performance of our NLP system, we annotated 6 additional EHR notes as unseen hold-out data for testing. Those 6 notes included a total of 2,345 sentences, of which only 64 sentences (2.7%) were positive.

**Sentence samples from our dataset with 2 positive bleeding samples and 2 negative bleeding samples. POS: positive bleeding sample sentence; NEG: negative bleeding sample sentence.**

POS 1: Patient was admitted with hematemesis and blood per rectum.

POS 2: Anticoagulation has been held on this patient secondary to recent gastrointestinal bleed.

NEG 1: She has done well with the warfarin with no further thromboembolic episodes and no bleeding problems.

NEG 2: The patient is also on Keppra for seizure activity, and he has been seizure-free on that medication.

### Experimental Setup

We implemented the neural network models in Pytorch [39]. For the evaluation metrics, we used precision, recall, and F-score and reported the overall performance as well as the performance on positive instances and negative instances. In addition, we reported the area under the receiver operating characteristic curve (AUC-ROC) score. The AUC-ROC plots the true positive rate (y-coordinate) against the false positive rate (x-coordinate) at various threshold settings. For the testing on the natural EHR notes, we reported overall accuracy as well as the precision, recall, and F-score on positive samples. All the word embedding sizes were initialized or pretrained with 200 dimensions. The dropout rate was set as 0.3, batch size as 16, and learning rate as 0.001. Optimization was performed using stochastic gradient descent. For the BiLSTM autoencoder model, the number of hidden neurons was 64.

### Models

We conducted experiments with the following baselines to compare them with our proposed model. We first compared our model with a strong traditional machine-learning classifier as the SVM model. The basic bag-of-words features are used for SVM. A stronger SVM baseline with both bag-of-words features and bag of the unified medical language system (UMLS) [40] concept features were implemented for comparison. In addition, as the proposed model was an integrated model including a word-embedding CNN and a BiLSTM autoencoder, we conducted experiments to determine the separate performance of the two components. Pretrained word embeddings were used in additional experiments to examine their effect in model performance.

#### Support Vector Machines+Bag of Words

A standard linear SVM classifier [41] with bag-of-words features was used. Parameter C (penalty parameter of the error term) was set as 1, and other parameters were set as default in the sklearn.SVM.SVC implementation [41].

#### Support Vector Machines+Bag of Words+Unified Medical Language System

A standard linear SVM classifier [41] with bag-of-words features and bag of UMLS [40] concept features were used. Further, we used MetaMap [42], a tool created by NLM that maps from free text to biomedical concepts in the UMLS, to identify medical phrases. The same parameters were used as mentioned in the Support Vector Machines+Bag of Words subsection above.

#### Autoencoder

The BiLSTM-based autoencoder model has been described above (see Methods section); all word embedding was randomly initialized and modified during training. A fully connected layer was used to connect the obtained hidden representation of 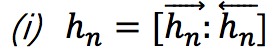
followed by a softmax operation to generate the prediction.

#### Autoencoder and Pretrained Word Embedding

This model was similar to the autoencoder model. However, in this model, we pretrained the word embedding on 4.7 million EHR notes using the Glove model [43]. The pretrained vectors were fine tuned for the task during training.

#### Convolutional Neural Network

The CNN model used has been described in the Methods section, with all word embeddings randomly initialized and modified during training.

#### Convolutional Neural Network and Pretrained Word Embedding

This model was similar to the CNN model. The same pretrained word embeddings described in the Autoencoder and Pretrained Word Embedding experiment were used, and the vectors were fine tuned for the task during training.

#### Hybrid Convolutional Neural Network and Long Short-Term Memory Autoencoder

Our proposed model incorporates CNN, pretrained word embedding, and a BiLSTM-based autoencoder, as described in the Methods section.

#### Convolutional Neural Network for Negation Bleeding

As negation bleeding (eg, NEG1 in Textbox 1) is a relatively difficult and misleading subset of the corpus for the model to make predictions, we conducted this extra experiment with only the 285 sentences that mentioned negated bleeding as negative samples. Of these, 185 sentences were used with the 1451 positive samples for training, and the remaining 100 sentences were used for testing.

#### Hybrid Convolutional Neural Network and Long Short-Term Memory Autoencoder for Negation Bleeding

The same data setting was used as mentioned above for Convolutional Neural Network for Negation Bleeding.

### Experimental Results

Our HCLA model showed the best performance across all evaluation metrics, with an AUC-ROC value of 0.957, overall F-score of 0.938, and F-scores of 0.932 and 0.943 for positive and negative sentences, respectively (Table 1). With pretrained word embedding, both the autoencoder and the CNN models performed better than learning word representation directly from the data. For the traditional SVM model, improved performance was achieved by incorporating the UMLS knowledge, leading to an overall F-score of 0.886 and an AUC-ROC value of 0.934 compared to an F-score of 0.862 and an AUC-ROC of 0.921 without the UMLS features. The incorporation of UMLS knowledge especially improved the precision score on positive samples with a large increase of 0.043. The precision score of all CNN models demonstrated a consistent increase in all positive and negative samples. As shown in Table 1, the CNN model without the autoencoder outperformed the model that was solely built on the autoencoder.

To further evaluate our model’s performance on natural EHR notes (as compared to negative sampling), we tested the proposed HCLA model on the 6 extra annotated notes. The resulting overall accuracy outcomes and precision, recall, and F-score on positive samples are shown in Table 2.

**Table 1:** Comparison of the study results at baseline.

Model	Positive			Negative			Overall			AUC-ROC^a^
	Precision	Recall	F-score	Precision	Recall	F-score	Precision	Recall	F-score	
SVM^b^+BOW^c^	0.848	0.883	0.865	0.878	0.841	0.859	0.862	0.862	0.862	0.921
SVM+BOW+UMLS^d^ concept	0.891	0.887	0.889	0.870	0.886	0.878	0.886	0.886	0.886	0.934
Autoencoder	0.861	0.855	0.858	0.856	0.862	0.859	0.859	0.859	0.859	0.920
Autoencoder+pretrained word embedding	0.875	0.869	0.872	0.870	0.876	0.873	0.872	0.872	0.872	0.926
CNN^e^	0.908	0.877	0.892	0.879	0.910	0.894	0.893	0.893	0.893	0.938
CNN+pretrained word embedding	0.930	0.911	0.920	0.912	0.931	0.921	0.921	0.921	0.921	0.946
HCLA^f^	0.954	0.912	0.932	0.925	0.961	0.943	0.938	0.938	0.938	0.957
CNN for negation bleeding	N/A^g^	N/A	N/A	0.820	0.820	0.820	N/A	N/A	N/A	N/A
HCLA for negation bleeding	N/A	N/A	N/A	0.860	0.860	0.860	N/A	N/A	N/A	N/A

^a^AUC-ROC: area under the receiver operating characteristic curve.

^b^SVM: support vector machines.

^c^BOW: bag of words.

^d^UMLS: unified medical language system.

^e^CNN: convolutional neural network.

^f^HCLA: hybrid convolutional neural network and long short-term memory autoencoder.

^g^N/A: not applicable.

**Table 2:** Performance of the hybrid convolutional neural network and long short-term memory autoencoder model on natural electronic health record notes.

Performance parameter	Value
Overall accuracy	0.938
Precision on positive samples	0.992
Recall on positive samples	0.944
F-score on positive samples	0.967

## Discussion

### Principal Findings

This study addresses the detection of important bleeding events in EHR notes. Clinical phenotyping is challenging mainly due to irregularity of clinical narratives, which incorporates domain-specific medical jargon, abbreviations, incorrect use of natural language (eg, spelling errors), etc [44,45]. In addition, negation is common in the clinical domain, which adds complexity. The difficulty of the NLP task is further exacerbated by the limited size of human annotated gold standard positive samples, which makes it difficult for our data-driven deep learning approaches to extract effective features. However, the proposed HCLA model (Textbox 1) achieved the best result of 0.938 (F-score) and 0.957 (AUC-ROC value).

Our results show that all end-to-end CNN models outperformed the baseline SVM model, despite the incorporation of knowledge-based features for SVM. When we pretrained word embedding over large unlabeled EHR notes (4.3 million), the overall F-score performance improved by approximately 0.03, demonstrating the effectiveness of using unlabeled data. Our results demonstrate that the BiLSTM-based autoencoder improved sentence representation. By concatenating the representation of the BiLSTM-based autoencoder, we further improved the performance by 0.017 (from 0.921 to 0.938) and the AUC-ROC value by 0.957, even though the model was trained with a relatively small data set (1451 positive samples and 285 negative samples).

Our annotated data incorporated both bleeding and negated bleeding events. Detection of bleeding signals is challenging from narratives, but separating true bleeding events from negated bleeding events is more challenging due to different negation variations. We therefore evaluated how our model performed in terms of accurately identifying negated bleeding events. Comparing the data setting described above for CNN and HCLA for negation bleeding, the CNN model achieved an accuracy of 0.82 and our proposed model achieved an accuracy of 0.86. The results validate the ability of the model to learn to grasp meaningful features in the dataset, rather than just depend on “bleeding”-related word indicators. For example, in Textbox 1, the NEG1 sentence contains the word “bleeding” but describes a nonbleeding event.

**Misclassified sentence samples by the proposed hybrid convolutional neural network and long short-term memory autoencoder system. NEG: negative bleeding sample sentence; POS: positive bleeding sample sentence.**

NEG1: Here is no chest pain, chest pressure, shortness breath, wheezing, coughing, nausea, vomiting, or gastrointestinal bleeding.

POS1: At that time, he was found to be anemic with heme-positive stools and was admitted for evaluation.

POS2: There is no evidence or stigmata for recent bleed.

Table 2 presents the model’s performance in real applications in the 6 natural EHR notes. In this unbalanced data of total 2345 sentences with only 64 positive sentences (2.7%), our model showed good results. The overall accuracy was 0.938. The model achieved high precision, recall, and F-score of 0.992, 0.944, and 0.967, respectively, on positive samples. Performance in positive samples is meaningful because it reflects how accurately the NLP system detects bleeding events in the EHR notes.

### Error Analysis

Our error analyses showed that HCLA needs improvement in negation detection and analyzing complex sentences. Textbox 2 includes representative examples to show that HCLA failed the classification. The first sentence in Textbox 2 was a negated bleeding sample, but was misclassified as a positive instance. Since our employed CNN model mainly focused on the local context, it may fail to recognize the distant negation cue “no.” The second sentence was a positive sample, but was misclassified as a negative sample by the system. The second sentence is complex and required knowledge inference, which may be challenging for the model.

The third sentence in Textbox 2 was annotated as a positive instance, but seemed to be correctly identified by our system as a negative instance. On examining the note, we found that it was a follow-up of a patient whose chief complaint was hemorrhoidal bleeding. Our annotators annotated bleeding events at the whole-note level. Although the bleeding event in this specific sentence in the section of Physical Examination, seemed to be negative, it was annotated as “bleeding present.” In this case, the annotators interpreted the bleeding as the present complaint, even though the sentence clearly indicated no evidence of a recent bleed.

This sentence represents a complex case of annotation consistency. The annotation guideline needs to be updated to refine the definition of “assertion.” On the other hand, this annotation highlights one limitation of our NLP system that is based on sentence-level classification. Our future work will focus on exploring the context of the whole note.

### Study Strength

The contributions of this study are several folds: This study is the first to automatically detect bleeding events from EHR notes. We have demonstrated the effectiveness of HCLA as a high-performance bleeding event-detection NLP system from EHRs. In addition, we have demonstrated the effectiveness of the HCLA architecture that can be trained from small annotated data.

### Limitations

We acknowledge a few limitations to this study. The gold dataset for our experiments was relatively small. Therefore, we built unsupervised models to leverage the large unlabeled EHR data in order to improve the performance. However, the generalizability and robustness of the model were not evaluated on a large scale. In addition, our system was based on sentence-level classification and does not consider the context of whole notes.

### Conclusions

This is the first study to address bleeding detection in EHRs. Our proposed HCLA neural network model effectively outperformed standard CNN models, autoencoder models, and SVM models by using a limited number of expert annotations. In the future, we will attempt to use active learning methods in order to improve the efficiency of experts’ annotation. Depending on more high-quality annotation, we will further mine data on bleeding causes, anatomic sites of bleeding, bleeding severity, and assertion (current vs history) from EHRs, as tasks are important and require further examination.

## Abbreviations

ADE: adverse drug events
AUC-ROC: area under the receiver operating characteristic curve
BiLSTM: bidirectional long short-term memory
BOW: bag of words
CNN: convolutional neural network
EHR: electronic health record
HCLA: hybrid CNN and LSTM autoencoder
LSTM: long short-term memory
NEG: negative bleeding sample sentence.
NLP: natural language processing
POS: positive bleeding sample sentence.
RNN: recurrent neural network
SVM: support vector machines
UMLS: unified medical language system
